# Self-Reported Omega-3 Supplement Use Moderates the Association between Age and Exercising Cerebral Blood Flow Velocity in Older Adults

**DOI:** 10.3390/nu12030697

**Published:** 2020-03-05

**Authors:** Carolyn S. Kaufman, Eric D. Vidoni, Jeffrey M. Burns, Mohammed R. Alwatban, Sandra A. Billinger

**Affiliations:** 1 Department of Molecular & Integrative Physiology, University of Kansas Medical Center, Kansas City, KS 66160, USA; ckaufman3@kumc.edu; 2 University of Kansas Alzheimer’s Disease Center, Fairway, KS 66103, USA; evidoni@kumc.edu (E.D.V.); jburns2@kumc.edu (J.M.B.); 3 Department of Physical Therapy and Rehabilitation Science, University of Kansas Medical Center, 3901 Rainbow Boulevard, MS 2002, Kansas City, KS 66160, USA; malwatban@kumc.edu

**Keywords:** vascular, omega-3, exercise, cerebral blood flow, middle cerebral artery, supplements

## Abstract

Cerebral blood flow (CBF) decreases across the lifespan, and chronic conditions such as dementia and stroke accelerate this decline. Impaired CBF results in reduced delivery of oxygen and nutrients, which can damage the brain over time. Thus, there is a need to identify lifestyle interventions, including diet and exercise, to maintain CBF with aging and in the presence of chronic disease. In the present study, we used transcranial Doppler ultrasound to record middle cerebral artery velocity (MCAv), a surrogate measure of CBF, during moderate-intensity exercise in sedentary, cognitively normal older adults (*n* = 90). A multiple linear regression model (*F*(4, 85) = 3.21, *p* = 0.02) showed that self-reported omega-3 supplement use significantly moderated the association between age and mean exercising MCAv in these individuals (*p* = 0.01). Older age was associated with lower exercising MCAv in the group not taking omega-3 supplements, while exercising MCAv showed no decline with increasing age in the group who reported omega-3 supplement use. These findings suggest omega-3 supplementation may have an important role in the preservation of CBF with aging.

## 1. Introduction

Evidence increasingly suggests that omega-3 polyunsaturated fatty acids promote vasodilation via improved endothelial function and relaxation of vascular smooth muscle cells [1]. These effects may impact the cerebral vasculature resulting in higher cerebral blood flow (CBF) [2,3]. For example, one double-blind, placebo-controlled study found that dietary supplementation with fish oil increased CBF during cognitive tasks in healthy young adults [4]. More recently, a randomized controlled trial in older adults with mild cognitive impairment showed that omega-3 fatty acid supplementation reduced cognitive and functional decline [5]. While this trial did not explore the mechanisms through which omega-3 consumption improved brain function, a previous study found that fish oil supplementation improved the CBF response to hypercapnia and to cognitive stimuli in older adults with borderline hypertension [6], suggesting that omega-3 may act mechanistically by improving blood flow to the brain. These findings are important since the brain receives 15–20% of cardiac output despite comprising only 2% of body mass [7]. This disproportionate blood supply is necessary in order to remove potentially harmful metabolic waste products and to provide crucial oxygen and nutrients to working neurons and glia [8]. It has been reported that resting cerebral blood flow (CBF) decreases over time during healthy aging [9], and this decline is accelerated in individuals developing cognitive impairment such as Alzheimer’s disease [10,11,12,13,14,15,16]. Additionally, our laboratory reported decreased CBF velocity both at rest and during exercise in people with chronic conditions such as atherosclerotic cardiovascular disease [17] and stroke [18,19]. Further, we found that cognitively normal older adults with elevated brain amyloid-β (Aβ) have a reduced CBF response during exercise compared to cognitively normal older adults with non-elevated brain Aβ [20], suggesting that the brain may receive less oxygen and fewer nutrients in people with dementia-associated pathology. Thus, there is a crucial need to identify methods of maintaining and/or augmenting CBF both at rest and during physiological challenges, such as exercise.

Lifestyle interventions, including healthy diet and regular physical activity, are important for maintaining optimal brain health [21], partially denoted by maintenance of CBF. Omega-3 fatty acid supplementation has been shown to increase CBF in response to carbon dioxide and during cognitive tasks [4,6]. However, the impact of omega-3 polyunsaturated fatty acid supplementation on CBF during other stimuli relevant to daily life, such as exercise, has yet to be elucidated. Thus, the aim of the current study was to analyze the connection between self-reported omega-3 supplementation and CBF velocity during exercise in older adults. We hypothesized that self-reported omega-3 supplementation would (1) be associated with higher middle cerebral artery velocity (MCAv), a surrogate measure of CBF, during moderate-intensity exercise, and (2) moderate the association between aging and MCAv during exercise.

## 2. Materials and Methods

### 2.1. Recruitment

This is a secondary analysis of a larger data set [17,20,22]. Participants were recruited through the University of Kansas (KU) Alzheimer’s Disease Center. Inclusion criteria were: (1) between 65 and 90 years of age, (2) underactive or sedentary lifestyle, (3) classified as cognitively normal/non-demented based on neuropsychological testing and a Clinical Dementia Rating (CDR) = 0, and (4) completion of [18F] a florbetapir positron emission tomography (PET) scan within six months of the vascular laboratory visit. Exclusion criteria were: (1) clinically significant depression or anxiety, (2) insulin-dependent diabetes, (3) Diagnostic and Statistical Manual of Mental Disorders-IV-defined drug or alcohol abuse within the prior two years, (4) acute decompensated congestive heart failure or class IV heart failure, (5) myocardial infarction or symptoms of coronary artery disease within the prior two years, (6) inability to exercise due to pain or restrictions set by physician, or (7) major orthopedic disability. Written informed consent was obtained from all participants. All study procedures were approved by the KU Institutional Review Board (IRB#: STUDY00001444, IRB ID: CR00005780) and complied with the Declaration of Helsinki.

### 2.2. Positron Emission Tomography (PET) Scan

PET images were obtained on a GE Discovery ST-16 PET/CT Scanner. Two 5-min PET brain frames were acquired continuously, approximately 50 min after [18F] florbetapir (370 MBq) administration. Self-adherent wrap across the forehead was used to minimize head movement. Frames were summed, and attenuation corrected. To increase tissue uptake specificity, we calculated standardized uptake value ratios (SUVR) in several individualized regions using a custom processing pipeline in SPM12 (http://www.fil.ion.ucl.ac.uk/spm) as described previously [23].

### 2.3. Vascular Laboratory Visit

Each participant completed a morning vascular laboratory visit described in detail in previous publications [17,20,22]. Briefly, a 5-lead electrocardiogram (Cardiocard, Nasiff Associates, Central Square, NY) recorded heart rate (HR). A finger plethysmograph (Finometer Pro, Finapres Medical Systems) was fitted to the middle finger of the left hand and collected continuous beat-to-beat blood pressure measurements, which were used to calculate mean arterial pressure (MAP). A nasal cannula connected to a capnograph (BCI Capnocheck 9004) continuously recorded end-tidal carbon dioxide (P_ET_CO_2_). A 2 MHz transcranial Doppler ultrasound (TCD) probe (RobotoC2MD, Multigon Industries) was placed over the temporal window in order to insonate the left middle cerebral artery (MCA). Gain, gate, and depth settings were adjusted to optimize the MCA signal using established practice standards for TCD [24,25]. TCD sonographers were blinded to participant demographics and amyloid status.

Moderate-intensity exercise was defined for each participant as 40–60% of the age-predicted HR reserve [26]. Maximum HR (HRmax) was determined by using either [27]:

HRmax = 220 − age, for participants not taking a beta-blocker (1)

HRmax = 164 − (0.72 ∗ age), for participants taking a beta-blocker (2)

The HR range for moderate-intensity exercise was determined using the Karvonen formula [26]:

HR range = [% exercise intensity ∗ (HRmax − resting HR)] + resting HR (3)

All participants maintained a step rate of approximately 90 steps per minute on a recumbent stepper (NuStep T5XR). Each participant began exercise at 40 Watts, and the resistance was increased until the individually determined target HR range was reached. Data collection began and continued until the participant completed 8 min of continuous exercise in the moderate-intensity HR range. Sampling frequency was 500 Hz using an analog-to-digital data acquisition board (National Instruments) and a custom script written for MATLAB (v2015, Mathworks). MAP, P_ET_CO_2_, and MCAv were resampled at 10 Hz. Mean MCAv during exercise was calculated for each participant as the average MCAv over the duration of the 8-min recording [17,20,22].

### 2.4. Demographic and Physiologic Characterization

Height and weight obtained at the vascular laboratory visit were used to calculate body mass index (BMI) [28]. Measurements obtained during the clinical visit were used to calculate the 10-year atherosclerotic cardiovascular disease (ASCVD) risk score using the Pooled Cohort Risk Assessment Equation provided by the American Heart Association and the American College of Cardiology Guideline on the Assessment of Cardiovascular Risk [29]. Education and medication/supplement use were self-reported at the visit.

### 2.5. Statistical Analyses

All statistical analyses were performed using SPSS Statistics (IBM). Between-group differences (self-reported omega-3 supplementation “yes” or “no”) were assessed using independent *t*-tests, Mann–Whitney *U*-tests, chi-square tests, or Fisher’s exact tests, as appropriate. We performed a multiple linear regression analysis to explore the potential effect of Aβ load, aging, self-reported omega-3 supplement use, and the interaction between aging and self-reported omega-3 supplement use on our primary outcome variable, mean MCAv during exercise. We set α = 0.05 to protect against type I error and did not correct for multiple comparisons due to the exploratory nature of this study.

## 3. Results

Of the 90 participants, sixty-two (69%) were female, and forty-six (51%) reported omega-3 supplement use. There were no differences between self-reported omega-3 supplementation “yes” or “no” groups in the available demographic factors (Table 1). There were also no significant differences between groups in target Watts, average P_ET_CO_2_, and average MAP during exercise, which influence exercising MCAv. Finally, there were no significant differences between groups in the proportion of individuals who reported taking vasoactive medications that could potentially influence blood flow, including anti-hypertensives and statins.

Our primary outcome measure was mean MCAv during exercise [17,18,19,20,22]. We observed no outliers in the data as assessed by inspection of a boxplot. Mean MCAv during exercise was normally distributed for each level of omega-3 supplementation as assessed by the Shapiro–Wilk test (*p* > 0.05). There was homogeneity of variances as assessed by the Levene’s test for equality of variances (*p* = 0.267). Data are mean ± standard deviation, unless otherwise stated. Contrary to our hypothesis, there was no significant difference (*p* = 0.590) in mean MCAv during exercise between participants reporting omega-3 supplement use (52.05 ± 10.8 cm/s) and participants reporting no omega-3 supplement use (50.71 ± 12.6 cm/s).

Next, a multiple regression was run to predict mean MCAv during exercise from Aβ load, self-reported omega-3 supplement use, age, and the interaction between self-reported omega-3 supplement use and age. We included Aβ load in the model to account for its potential confounding effect due to our previous finding that elevated Aβ load is associated with decreased MCAv response during exercise [20]. Independent variables were centered to reduce multicollinearity. Linearity was established by visual inspection of a scatterplot, and there was no evidence of multicollinearity as evidenced by no tolerance values less than 0.959. There were no outliers detected. There was homoscedasticity as assessed by visual inspection of the studentized residuals plotted against the predicted values. The studentized residuals were normally distributed as assessed by the Shapiro-Wilk test (*p* > 0.05). The multiple regression model significantly predicted mean MCAv during exercise, *F*(4, 85) = 3.208, *p* = 0.017, adjusted *R^2^* = 0.09. Regression coefficients and standard errors are found in Table 2.

**Figure 1:**
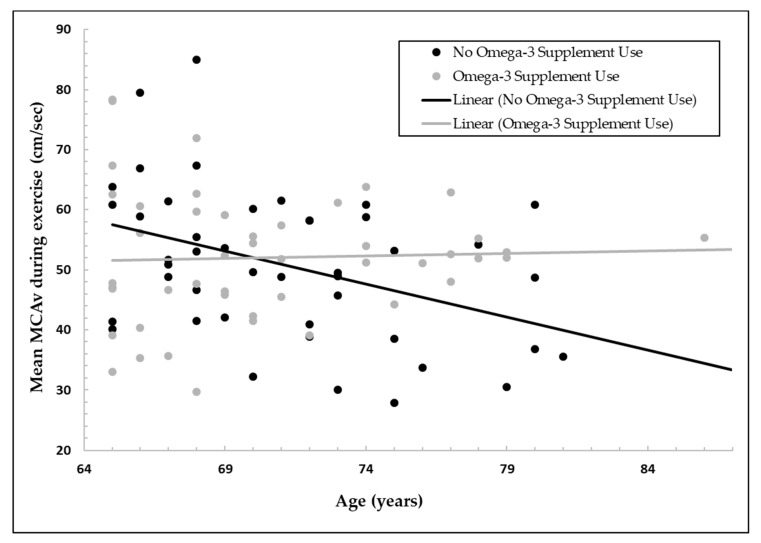
Middle cerebral artery velocity (MCAv; centimeters per second) during moderate-intensity exercise is shown as a function of age (years) for participants who reported omega-3 supplement use or no omega-3 supplement use. For the group not taking omega-3 supplements, increasing age was associated with a lower exercising MCAv, a surrogate measure of cerebral blood flow (CBF). In contrast, exercising MCAv was stable with increasing age in the group who reported taking omega-3 supplements.

Additionally, a hierarchical multiple regression was run to assess the increase in variation explained by the addition of the interaction term between omega-3 supplementation and age to the main effects model. Omega-3 supplementation moderated the effect of age on mean MCAv during exercise as evidenced by a statistically significant increase in total variation explained of 6.5%, *F*(1, 85) = 6.384, *p* = 0.013. Figure 1 shows a scatterplot of mean MCAv during exercise as a function of age for participants who reported taking or not taking omega-3 supplements.

## 4. Discussion

The major finding of this secondary analysis was that self-reported omega-3 supplementation significantly moderated the effect of age on mean MCAv during exercise. Specifically, there was a decline in exercising MCAv (a surrogate measure of CBF) with increasing age in the group not taking omega-3 supplements, while this age-related decline was not observed in the group reporting omega-3 supplement use. These results suggest that omega-3 supplementation may preserve CBF with aging during physiological challenges, such as moderate-intensity exercise, thus maintaining delivery of crucial oxygen and nutrients to the working brain. To our knowledge, we are the first to report this potential connection between omega-3 supplementation and improved CBF measures during exercise. However, this finding is consistent with randomized controlled trials that have found that omega-3 supplementation can increase CBF during other cerebrovascular challenges such as hypercapnia and cognitive tasks [4,6].

Both animal and human studies have shown that omega-3 fatty acids improve systemic vascular function [1,30], which could explain the preservation of CBF during exercise observed in the current study. For example, omega-3 fatty acids increase nitric oxide availability [31,32] and directly promote relaxation of vascular smooth muscle cells [33,34] resulting in vasodilation and increased blood flow. Additionally, a meta-analysis of randomized controlled trials found omega-3 supplementation decreased arterial stiffness in adults [35]. Finally, omega-3 fatty acids have anti-inflammatory and anti-oxidant properties [36,37,38], both of which could contribute to improved cerebrovascular health and higher blood flow during exercise in the older adults in the present study who reported taking supplements.

Regardless of the mechanism through which omega-3 supplement use stabilizes CBF, the consequent increased delivery of oxygen and nutrients to the brain could explain findings from other studies that show omega-3 fatty acids augment cognitive function and prevent cognitive decline [39,40,41,42]. For example, the connection between atherosclerosis and dementia has been well-established [43], and a meta-analysis of randomized controlled trials showed that omega-3 fatty acids significantly slowed the progression of atherosclerosis [44], suggesting that omega-3 fatty acids could potentially prevent cognitive decline through improvements in vascular health. One recent trial showed that six months of supplementation with omega-3 and omega-6 fatty acids in combination with antioxidant vitamins reduced both cognitive and functional decline in adults with mild cognitive impairment [5]. Although mechanistic pathways were outside the scope of the secondary analysis, the authors suggested that omega fatty acids may have acted partially through improvements in endothelial function [5]. Indeed, the observed improvement in functional capacity, including an increase in distance walked on the 6-min walk test, coupled with enhanced cognitive function [5] points to a systemic effect of supplementation, which could be explained by whole-body improvement in vascular function that includes higher CBF. Future studies that measure changes in CBF would provide evidence for this mechanistic pathway. Importantly, one randomized trial lends credence to this potential mechanism by showing that 20 weeks of omega-3 supplementation increased CBF response to carbon dioxide and cognitive tasks in older adults with borderline hypertension [6]. The current study expands upon these findings by demonstrating preserved exercising CBF velocity with age with omega-3 supplementation, specifically in older adults who are at increased risk for vascular dysfunction. Taken together, the data from the current study and those mentioned above point to a potential role for omega-3 fatty acids in improving cerebrovascular function with the goal of delaying cognitive decline or dementia.

We found no significant group difference in mean exercising MCAv by omega-3 supplement use. Rather, significant group differences were observed in the relationship between aging and exercising MCAv (*p* = 0.013) with the group taking omega-3 supplements having stable exercising MCAv with increasing age in contrast to the group not taking omega-3 supplements who had lower exercising MCAv with older age. This suggests that omega-3 supplementation could be particularly beneficial for maintaining CBF with age.

To our knowledge, the present study was the first to explore the connection between omega-3 supplement use and CBF velocity during exercise. Importantly, the exercise stimulus utilized was similar in intensity to walking up a flight of stairs [26] and thus reflective of physiological challenges incurred daily by the cerebral vasculature in these cognitively normal older adults. Impaired CBF metrics during exercise may reflect cerebrovascular dysfunction and brain pathology, as we have previously demonstrated a decreased CBF velocity response to exercise in individuals after ischemic stroke [18,19], with Alzheimer’s-associated brain pathology [20], and in disease-free aging [45]. In the current study, we report data that suggest that supplementation with omega-3 fatty acids may be one potential avenue to counteract this decline in exercising CBF velocity, which could in turn improve brain health and function.

This secondary analysis has several limitations. Importantly, omega-3 supplement use was self-reported in these individuals. Thus, these findings should be considered hypothesis-generating and could be used to justify future studies that would yield more conclusive results. For example, a future blinded, randomized, controlled trial would allow for more robust conclusions about the causative effects of omega-3 on CBF. Additionally, we recorded omega-3 supplement use as either “yes” or “no”, which does not account for potential dosage effects or the influence of different brands of supplements. We also did not measure participant diet, which could be an alternative source of omega-3 fatty acids. The data are cross-sectional; this limits interpretation of the relationship between aging and MCAv. Future longitudinal studies are necessary to characterize the effects of omega-3 supplementation within the same individuals over time. We did not characterize hematocrit levels in the participants, which could affect the blood hemodynamic properties and should be considered in future work. The linear regression model, although significant (*p* = 0.017), accounted for only 9% of the variance of the dependent variable, mean MCAv during exercise (*R^2^* = 0.09). Clearly, there are other factors that account for differences in CBF during exercise, likely including both modifiable factors, such as physical activity level, and non-modifiable factors, such as genetic background [21]. It is therefore important to acknowledge that omega-3 supplementation may be just one of many important components that influence CBF during exercise. We considered the possibility that participants taking omega-3 supplements may have a higher rate of use of other vasoactive medications and/or a healthier biomedical profile, which could confound the results. We attempted to address these concerns by comparing medication use and quantifiable health metrics, such as BMI and ASCVD risk score, between the two groups and found no significant differences between those taking or not taking omega-3 supplements (Table 1). Finally, we utilized TCD in the present study, because it is currently the only practical method for indexing CBF simultaneously with exercise [46], as other popular methods for measuring CBF, such as arterial spin labeling, require participants to lie stationary inside a magnetic resonance imaging scanner. However, we acknowledge that using MCAv as a surrogate measure of flow necessitates the assumption of constant MCA diameter. Whether the MCA undergoes changes in diameter is a matter of dispute [47,48], and any diameter changes are likely negligible during the moderate-intensity exercise stimulus used in this study [49,50,51,52].

The present study found that self-reported omega-3 supplement use moderates the relationship between aging and MCAv during exercise in cognitively normal older adults. Specifically, MCAv during exercise was relatively stable with aging in the group who reported taking omega-3 supplements, while MCAv during exercise was lower with increasing age in the group who did not report taking omega-3 supplements. These results suggest that omega-3 supplement use might improve CBF during physiological challenges such as exercise. This may be important for maintaining adequate delivery of oxygen and nutrients to the brain over time, which could contribute to the prevention of cognitive decline. Future longitudinal, randomized, controlled trials are warranted to explore these relationships.

## Figures and Tables

**Table 1:** Demographics, physiological measurements, and medication use.

	Omega-3 “No” (*n* = 44)	Omega-3 “Yes” (*n* = 46)	All Participants (*n* = 90)	*p*
Sex, *n* female [% female]	33 [75%]	29 [63%]	62 [69%]	0.221
Age, years	71.2 [4.6]	70.5 [5.0]	70.8 [4.8]	0.335
Education, years	16.8 [2.7]	16.6 [2.6]	16.7 [2.6]	0.738
ASCVD Risk Score, %	16.2 [10.3]	15.8 [8.9]	16.0 [9.5]	0.837
Body Mass Index, kg/m^2^	26.2 [4.3]	27.4 [4.2]	26.8 [4.3]	0.083
Amyloid-β (Aβ) Load, SUVR	1.04 [0.17]	1.02 [0.15]	1.03 [0.16]	0.994
Target Exercising Watts	58.3 [21.4]	65.1 [21.9]	61.8 [21.8]	0.136
Exercising P_ET_CO_2_, mmHg	38.2 [4.1]	37.3 [4.2]	37.7 [4.2]	0.286
Exercising MAP, mmHg	105.7 [24.1]	103.3 [15.8]	104.5 [20.3]	0.948
ACE inhibitor use, *n* [%]	3 [7%]	6 [13%]	9 [10%]	0.486
ARB use, *n* [%]	6 [14%]	8 [17%]	14 [16%]	0.623
Beta-blocker use, *n* [%]	6 [14%]	6 [13%]	12 [13%]	0.934
CCB use, *n* [%]	4 [9%]	7 [15%]	11 [12%]	0.375
Thiazide use, *n* [%]	1 [2%]	1 [2%]	2 [2%]	1.000
Statin use, *n* [%]	17 [39%]	23 [50%]	40 [44%]	0.278

Values are mean [standard deviation] unless otherwise noted. ASCVD Risk Score = atherosclerotic cardiovascular disease risk score; SUVR = standard uptake value ratio, arbitrary units; Exercising P_ET_CO_2_ = average end-tidal carbon dioxide during exercise; Exercising MAP = average mean arterial pressure during exercise; ACE inhibitor = angiotensin-converting-enzyme inhibitor; ARB = angiotensin II receptor blocker; CCB = calcium channel blocker.

**Table 2:** Summary of multiple regression analysis results (*n* = 90).

Variable	*B*	SE*_B_*	β	*p*-Value
Intercept	66.306	7.893		0.000 *
Amyloid-β (Aβ) Load	−14.262	7.588	−0.193	0.064
Omega-3 supplement use	0.689	2.358	0.030	0.771
Age	−0.436	0.250	−0.180	0.085
Omega-3 supplement use × Age	1.253	0.496	0.258	0.013 *

The dependent variable predicted by the regression model was mean middle cerebral artery velocity during moderate-intensity exercise (exercising MCAv). B = unstandardized regression coefficient; SE_B_ = standard error of the coefficient; β = standardized coefficient; * significant (*p* < 0.05).
